# Permanent wettability of a novel, nanoengineered, clinically available, hyaluronan‐coated dental implant

**DOI:** 10.1002/cre2.130

**Published:** 2018-09-05

**Authors:** Marco Morra, Clara Cassinelli, Elisa Torre, Giorgio Iviglia

**Affiliations:** ^1^ Nobil Bio Ricerche srl Italy

**Keywords:** dental implants, hydrophilicity, wettability

## Abstract

The objectives of this study are to evaluate long‐term wettability of novel surface‐engineered, clinically available dental implants, featuring a surface nanolayer of covalently linked hyaluronan, and to confirm the relationships between wetting properties and surface nanostructure and microstructure. Wettability measurements were performed on clinically available hyaluronan‐coated Grade 4 titanium implants, packaged and sterile, that is, in the “on the shelf” condition, after 1 year from production. Wetting properties were measured by the Wilhelmy plate method. Analysis of the surface structure and chemistry was perfomed by X‐ray photoelectron spectroscopy (XPS), scanning electron microscopy (SEM) and energy‐dispersive X‐ray (EDX) analysis, atomic force microscopy (AFM), and *ζ*‐potential measurement, either on implants or disks or plates subjected to the same surface‐engineering process. Results show that hydrophilicity and ensuing capillary rise of the hyaluronan‐coated implant surface is unaffected by aging and dry storage. Chemical analysis of the implant surface by XPS and evaluation of the *ζ* potential indicate that hyaluronan chemistry and not that of titanium dictates interfacial properties. Comparison between XPS versus EDX and SEM versus AFM data confirm that the thickness of the hyaluronan surface layer is within the nanometer range. Data show that nanoengineering of the implant surface by linking of the hydrophilic hyaluronan molecule endows tested titanium implants by permanent wettability, without need of wet storage as presently performed to keep long‐term hydrophilic implant surfaces. From an analytical point of view, the introduction in routine clinical practice of nanoengineered implant surfaces requires upgrading of analytical methods to the nanoscale.

## 1 INTRODUCTION

Ever since the expression of the Lampert rule for blood coagulation (Neubauer & Lampert, 1930), significant research effort has been devoted to the investigation of putative relationships between the deceivingly simple materials surface property called “wettability” and cell/tissue biological response (see Vogler, 2001, for a highly recommended review on the water wetting terminology and its usage in biomaterials science, and Rupp et al., 2014, for specific discussion on wettability of dental implant surfaces). Oral implantology did not escape this trend. Based on in vitro and in vivo evidences of better periimplant bone regeneration on high energy, or hydrophilic, titanium surfaces (Buser et al., 2004; Gittens et al., 2013; Park et al., 2012; Schwarz et al., 2007; Wennerberg et al., 2014), the SLActive concept was introduced in clinical practice (Chambrone, Shibli, Mercúrio, Cardoso, & Preshaw, 2015). The SLActive approach involves packaging of dental implants in saline solution, to preserve the freshly prepared microrough surface from contact with the atmosphere. It is based on knowledge developed by a prominent founding father of biomaterials surface science, Bob Baier, who discussed the concept of storage in water to preserve high‐energy surfaces of titanium dental implants in the 1980s and early 1990s (Baier & Meyer, 1988).

From a surface chemico‐physical point of view, wettability of titanium surfaces is pretty well understood. Titanium, or the titanium oxide surface layer produced on contact with atmospheric oxygen, belongs, as almost every metal or ceramic materials, to the domain of “high‐energy surfaces” (Zisman, 1964). In practice, their internal cohesive energy leads to an excess specific surface free energy some order of magnitude higher as compared to that of water. As a consequence, every pristine metal or ceramic surface, titanium and titanium oxide included, is fully wettable by water, or hydrophilic. The very same high surface energy, however, promotes adsorption of low‐energy ubiquitous hydrocarbons from the atmosphere. In practice, every metal or ceramic surface exposed to the atmosphere becomes hydrocarbon rich, in a time‐dependent way, turning from a high‐energy to a low‐energy surface, whose specific surface free energy is of the same order of magnitude, and in general lower, as compared with that of water. Hence, the finite water contact angles, or hydrophobicity, detected on real‐world titanium surfaces. In the case of titanium dental implants, the basic determinant of excess specific surface free energy, that is, the surface chemical composition, combines with the other properties that affect macroscopic wetting behavior and capillary rise, that is surface microtopography and thread size and shape (Rupp, Scheideler, Rehbein, Axmann, & Gels‐Gerstorfer, 2004), yielding increasingly hydrophobic surfaces as time from production goes bay. This behavior has been defined “biological aging” (Att et al., 2009; Choi et al., 2017a, 2017b), with a view to its biological effects. Its cause, however, is plainly chemical–physical, as just discussed. The hitherto most investigated chemical–physical approaches to exploit biological responses driven by hydrophilic implant surfaces involve preventing hydrocarbon adsorption by packaging in water (i.e., the already quoted SLActive approach) or getting rid of adsorbed hydrocarbons by UV light or glow discharge cleaning just before usage by side‐chair devices (Canullo et al., 2016; Choi et al., 2017a, 2017b).

Advances in nanotechnology and surface engineering offer further keys to hydrophilic implant surfaces: Wetting phenomena are exquisitely surface sensitive, that is, they are driven by the chemistry of the outermost atomic or molecular layer (Adamson & Gast, 1997). Thus, an intrinsically hydrophilic, adsorption‐resistant, nanometer‐thin, chemically linked surface layer, provided of course it does not adversely affect osteointegration, can impart permanent wettability to titanium implant surfaces, with no need of wet‐storage or hydrocarbon removal to restore hydrophilicity. Moreover, due to its nanosize, it does not affect implant surface microtopography and it is not subjected to massive delamination, as reported in the days of micrometer‐thick hydroxylapatite coatings.

Biomolecular nanoengineering of implant surfaces recently entered clinical practice, through novel dental implants featuring a surface nanolayer of permanently linked hyaluronan, or hyaluronic acid (HY; Morra et al., 2018). The relevance of HY molecular interactions and signaling properties for tissue regeneration and osteointegration, as well as references to relevant in vitro and in vivo works, can be found in the quoted paper. Besides, HY is also a paradigmatic hydrophilic biomolecule, and many of its current applications in medicine are based on its hydration properties (Knopf‐Marques et al., 2016; Morra, 2005; Sudha & Rose, 2014). On the light of claimed role of surface hydrophilicity on periimplant tissue response, it is of interest to investigate the effect of the surface‐linked hydrophilic HY nanolayer on implant wetting properties.

The scopes of the present work are the following: to evaluate long‐term wettability (>1 year on the shelf after production) of a novel surface‐engineered, clinically available dental implant, featuring a surface nanolayer of permanently linked HY; to confirm the relationships between wetting properties and surface nanostructure and microstructure; and to highlight the increasing role of finely tuned surface‐sensitive analytical techniques and relevant chemical–physical background in the understanding of implant clinical response, on the light of quick advancements of dental implants surface technology towards nanoengineering.

## 2 METHODS AND MATERIALS

**Figure 1:**
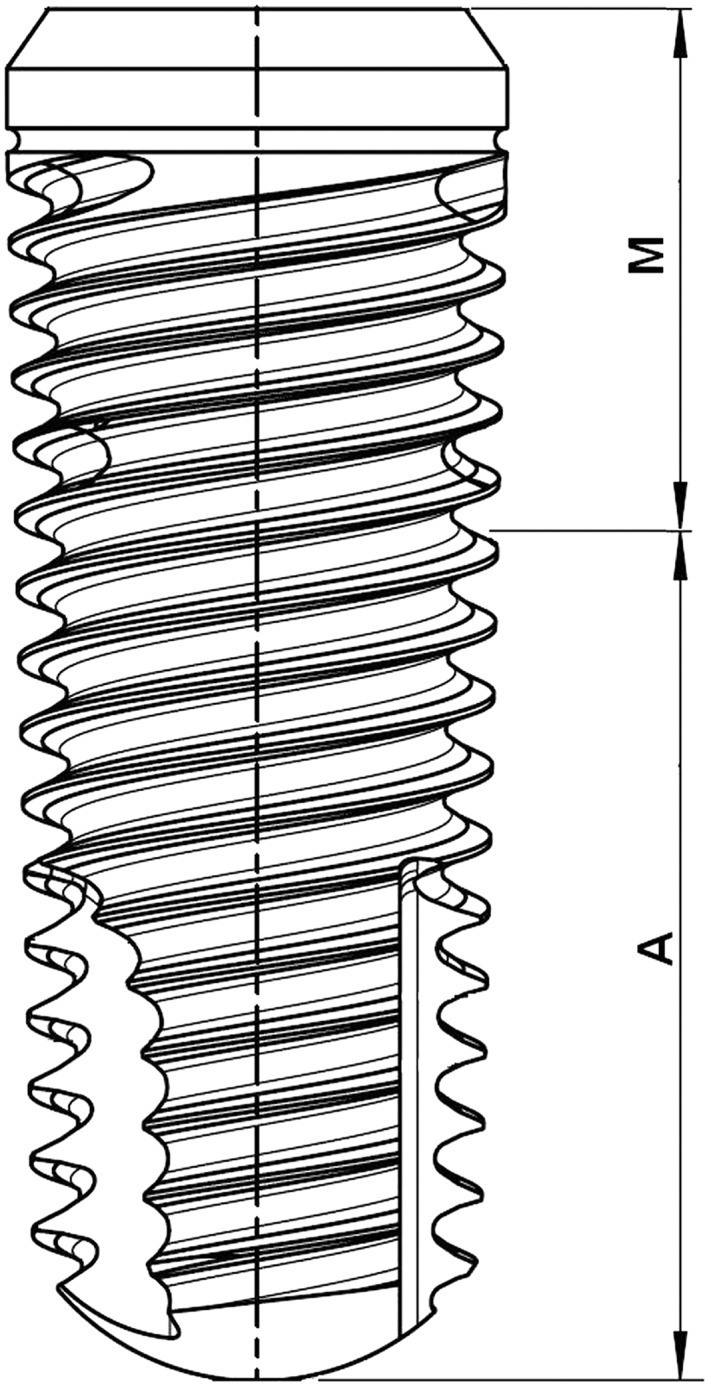
Drawing of the tested implant. The portion M is machined; the portion A is sandblasted and doubly acid etched

Measurements were performed on 4.1 × 16 Shapeone B HYHA implants produced by iRES SAGL, Riva di Caccia, Lugano, Switzerland (ref. code S1B4116HYHA). The macrodesign of the implant is shown in Figure 1. It belongs to the class of “hybrid” implants, meaning that, depending on the implant length, approximately 2/5 of the upper threaded portion is machined and the lower 3/5 portion is sandblasted and acid etched. Moreover, the whole implant surface is further coated by a nanolayer of HY, as described in Morra et al., 2018.

Tested implants were chosen in order to evaluate the effect of aging on wetting properties. Thus, implants with approximately 1 year and a few month aging (Lot 20173288 and Lot 20174409, respectively) were taken from the shelf. Implants were ready for clinical use that is packaged and sterile. The following measurements were performed:

### 2.1 Wettability

Wetting measurements were performed by a DCA 400 tensiometer (Gibertini, Milano, Italy), using a 60‐μm/s stage speed. This measurement is based on the Wilhelmy plate experiment (Rupp et al., 2014). Shortly, a microbalance measures the force exerted on a solid (the implant, in the present case) as it is immersed in a liquid (Milliq water in the present experiments). The total force can be written as

(1) $$F=W+\gamma_{L}\text{pcoϑ}+F_{b},$$

where *W* is the weight of the sample (i.e., its mass times the gravitational acceleration), *γ*_L_ the surface tension of the wetting liquid, *p* the sample perimeter, and *F*_b_ the buoyancy. Because *W* can be measured by the microbalance, *F*_b_ can be zeroed by extrapolation to zero depth of immersion and *p* can be measured independently; Equation (1) is commonly used to calculate the contact angle *ϑ* of a liquid of known surface tension *γ*_L_ on a solid of known perimeter *p*, or to measure the surface tension *γ*_L_ of a liquid that completely wets (*ϑ* = 0, cos *ϑ* = 1) a solid of known perimeter p.

In the present case, as widely described in the relevant section, the Wilhelmy plate experiments can be used just for qualitative purposes, for the following reasons: (a) the sample perimeter p is not constant, and it is a function of implant geometry and immersion depth; (b) the solid–liquid wetting interaction is dictated not just by the equilibrium contact angle *ϑ* (Rupp et al., 2014) but also by the implant surface microtopography, thread geometry, and screw pitch. Shortly, a dental implant is a highly unideal subject; measurements intended to extract contact angle data should be performed on cylinders or regularly shaped samples. Taking these considerations into account, the Wilhelmy plate experiment is however fit to show the qualitative outcome of wetting interactions and capillary phenomena, as described in Section 3.

### 2.2 Scanning electron microscopy and energy‐dispersive X‐ray analysis

The surface topography of the implants was evaluated by scanning electron microscopy (SEM). Analysis was conducted using an EVO MA 10 SEM (Carl Zeiss Microscopy GmbH, Jena, Germany). The electron acceleration voltage was maintained at 20 kV, the working distance between 10 and 12.5 mm. These parameters are reported in the images, along with the level of magnification (MAG) and the kind of detector utilized (Signal A = SE1 or CZ BSD).

Energy‐dispersive X‐ray (EDX) analysis was performed using an Aztec microprobe (Oxford Analytica), equipped with an X‐act X‐ray detector, enabling a resolution better than 129 eV.

### 2.3 X‐ray photoelectron spectroscopy

X‐ray photoelectron spectroscopy (XPS) analysis was performed using a Perkin Elmer PHI 5600 ESCA system (PerkinElmer Inc., Waltham, Massachusetts, USA). The instrument is equipped with a monochromatized Al anode operating at 10 kV and 200 W. The diameter of the analyzed spot is approximately 500 μm; the analyzed depth about 5 nm. The base pressure was maintained at 10–8 Pa. The angle between the electron analyzer and the sample surface was 45°. Analysis was performed by acquiring wide‐range survey spectra (0–1,000 eV binding energy) and detailed high‐resolution peaks of relevant elements. Quantification of elements was accomplished using the software and sensitivity factors supplied by the manufacturer. High‐resolution C1s peaks were acquired using a pass energy of 11.75 eV and a resolution of 0.100 eV/step.

Beside the just reported evaluation, the following test were made on disks or plates subjected to the same HY coating process.

### 2.4 Atomic force microscopy

Atomic force microscopy (AFM) was used to explore the surface nanotopography of 5 mm diameter machined Grade 4 Ti disks, both uncoated and HY coated. Measurements were performed using an NX10 Park AFM instrument (Park System, Suwon, Korea), equipped with 20‐bit closed‐loop XY and Z flexure scanners and a noncontact cantilever PPP‐NCHR 5M. This instrument implements a True Non‐contact™ mode, allowing minimization of the tip–sample interaction, resulting in tip preservation, negligible sample nanotopography modification and reduction of artifacts. On each sample, four different sample size areas were analyzed (20 × 20, 10 × 10, 5 × 5, and 1 × 1 μm) at a scan rate of 0.1 Hz.

### 2.5 *ζ*‐potential measurement

*ζ*‐potential measurements were performed using SurPass 3, equipped with an adjustable gap cell (Anton‐Paar GmbH, Graz, Austria). Measurements were conducted on 20 × 10‐mm Ti plates (Titanium foil 0.25 thickness from Sigma Aldrich), either uncoated or HY coated. The streaming channel was created by adjusting the distance between the plates surface in the adjustable gap cell, using 100% of the total surface area for measurement. Measurements were performed in a 0.001‐M KCl solution, according to *pH scan* method. It consists of the measurement of streaming current at different pH, between 2.8 and 8.2. The pH of electrolyte solution was modified automatically by the instruments using a 0.05‐mol/L HCl and 0.05‐mol/L NaOH. At each pH point, three measurements were performed in order to conditioning the sample, then the fourth value was taken and reported. Streaming potential and streaming current measurements are known to be useful methods for the investigation of charge displacement in the electrical double layer caused by an external force shifting the liquid phase tangentially against the solid (Luxbacher, 2014).

The *ζ*‐potential value is referred to the approach developed by Helmholtz and Smoluchowski:

(2) $$\zeta =\frac{{\mathrm{dI}}_{\mathrm{str}}}{d\Delta_{p}}*\frac{n}{\varepsilon *\varepsilon_{0}}*\frac{L}{A},$$

In this equation, $\frac{dI_{\mathrm{str}}}{d{∆}_{p}}$ coefficient is the streaming current, *L* is the length of the slit channel formed between the two samples forming the capillary, and *A* is the area cross section of the capillary. *η* and *ε* are the viscosity and dielectric coefficient of the solution forced to pass through the capillary. If we use the Ohm's law *I*_*str*_ *= U*_*str*_ */R* (where *R* is the electrical resistance inside the streaming channel and *U*_*str*_ is the potential) Equation (2) became

(3) $$\zeta =\frac{{\mathrm{dU}}_{\mathrm{str}}}{d\Delta_{p}}*\frac{n}{\varepsilon *\varepsilon_{0}}*\frac{L}{A}*\frac{1}{R}.$$

However, if the solid sample contributes to the conductance inside the streaming channel (i.e., metals), the calculation of streaming potential provides an apparent value of zeta potential, because the measurement of the cell resistance *R* is affected. Furthermore, the application of Helmholtz and Smoluchowski approach is possible just with planar solids surface, as in our case, since needs an exact knowledge about the geometry of the streaming channel, that is, the cell constant *L*/*A*. For the rectangular titanium slit, the length *L* and width are determined by the solid sample size; instead, the gap height is calculated from the measured volume flow rate of liquid passing through the streaming channel and the generated differential pressure.

All calculations were performed by the instrument software.

## 3 RESULTS

### 3.1 Wettability

**Figure 2:**
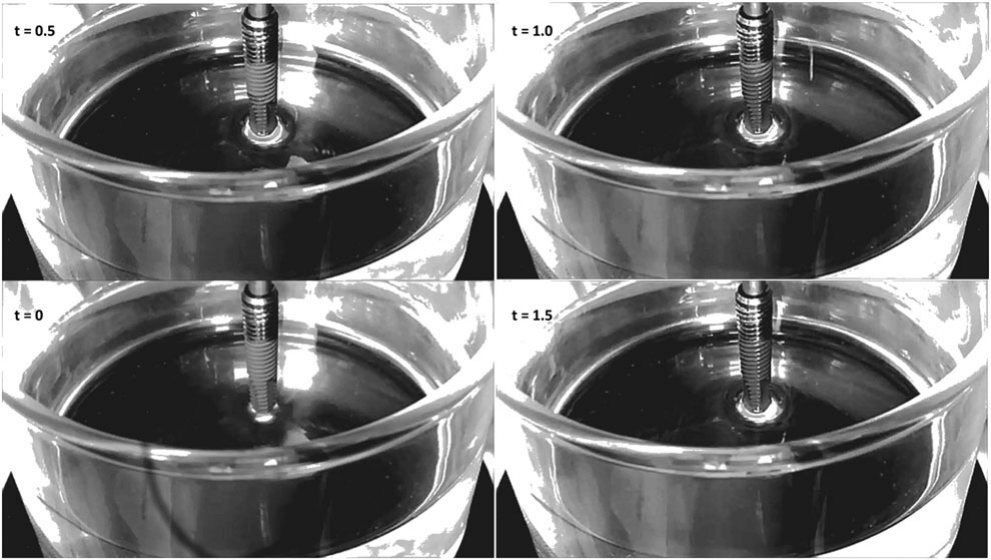
Timeframe image of the Wilhelmy plate experiment performed to evaluate capillary rise. Experimental time is reported in the frames

Figure 2 shows macroscopic evidence gathered from Wilhelmy plate measurements of the tested implants. In particular, the image shows a 1.5‐s timeframe sequence, that is, the time interval between two subsequent frames is 0.5 s, gathered on Lot 20173288 implants. The implant is hanged to the microbalance through a threaded connector and suspended over a slowly (60 μm/s) rising becker filled with MilliQ water (measured surface tension 71.4 mJ/m^2^), that is, from the beginning to the end of the timeframe sequence of Figure 2, the becker rises by 90 μm. At *t* = 0, the implant is still completely dry; the lower matt microrough texture and the upper shiny machined finish can clearly be appreciated. As soon as the apex touches water (or, more properly, as soon as it comes within the range of solid/liquid interfacial forces), a fast rise occurs within the capillary system made up by the thread and controlled by implant surface chemistry and roughness. After 0.5 s, water rises at about one half of the 10.5‐mm microrough portion; after further 0.5 s, the microrough portion is completely wet. At *t* = 1.5 s, capillary rise is moving towards the implant platform, about 16 mm away from the water level. Despite the 1 year aging on the shelf, the implant surface shows a highly hydrophilic behavior and it is fully wetted by water.

**Figure 3:**
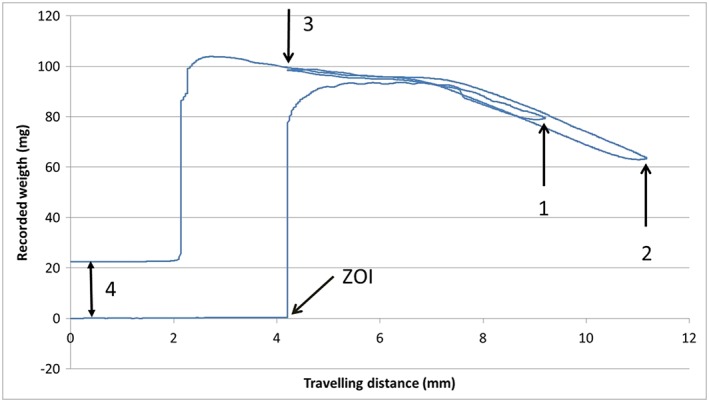
Force versus distance wetting loops for the tested implant. ZOI: zero depth of immersion. The implant is first immersed for 5 mm in water (to Point 1), then withdrawn up to Point 2, then immersed again for 7 mm (Point 3), then completely withdrawn

The same behavior is qualitatively captured by the outcome of the Wilhelmy plate measurement, that is, force versus distance wetting loops (Rupp et al., 2014). Figure 3 shows the recorded force on two subsequent loops, as follows: the implant touches water at zero depth of immersion. Immediately, a strong positive force (Equation (1), where *W* is zeroed by the software and *F*_b_ is still negligibly small) is exerted on the implant perimeter by the surface tension of water, operating at full force (cos *ϑ* = 1). The implant is first immersed for 5 mm in water (to Point 1), then withdrawn up to Point 2, then immersed again for 7 mm (Point 3), then completely withdrawn. No hysteresis is observed (i.e., differences between force recorded on advancing and receding, Rupp et al., 2004) because the surface is fully wet and the water front both on advancing and on receding slides over a water layer. Note that, while according to Equation (1), the recorded force is a function of the sample perimeter, hence, it should be higher on thread apex and lower in valleys; no evidence of force modulation by threads is detected in the graph of Figure 3. This is because threads are filled by water draw by capillary rise and the water front “feels” an apparent constant perimeter. Further note, on implant withdrawal, the remarkable increase of weight (double‐headed arrow, Point 4 in Figure 3) due to water captured within threads and surface microroughness. Altogether, both Figures 2 and 3 indicate a totally hydrophilic behavior and no evidence of aging on dry storage. The surface chemico‐physical basis behind these results is investigated in the forthcoming sections.

### 3.2 Scanning electron microscopy

**Figure 4:**
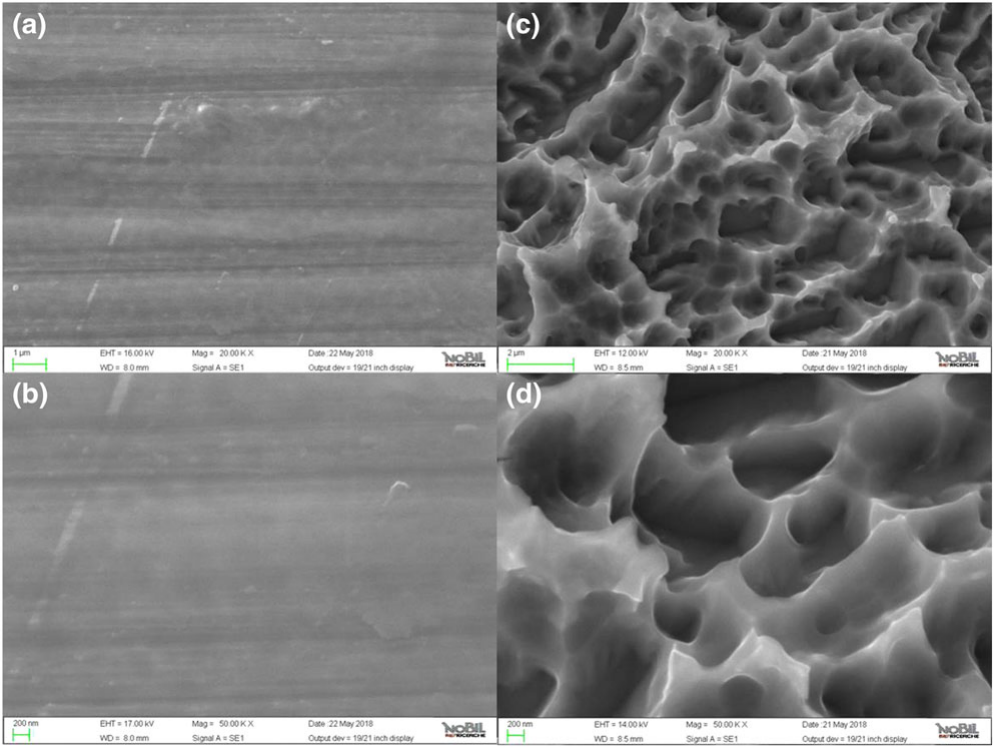
Scanning electron microscopy images of the tested implant surface. (a) machined portion 20,000×; (b) machined portion 50,000×; (c) sandblasted, doubly acid etched portion 20,000×; (d) sandblasted, doubly acid etched portion 50,000×

SEM and associated EDX analysis have been widely used in the evaluation of surface topography and chemistry of titanium dental implants. In the present case, most significant results are reported in Figure 4 that shows 20,000× and 50,000× images of the implant surface, both in the machined (a,b) and microrough (c,d) sections. Even if the trained eye can infer some evidence of hierarchical structure, both sections are still clearly dominated by the typical features of the respective microarchitectures: the parallel groves “fingerprints” of machining tools in the machined section (a,b), and the sharp, closely packed peaks, obtained by double acid‐etching in the microrough section (c,d). Note that, at this level of magnification, the other typical feature of sandblasted‐acid etched surfaces, that is, the macroroughness, due to blasting, cannot be appreciated because the field of view is too small. In other words, craters due to blasting are bigger than the approximately 15 × 10, or 6 × 4‐μm areas shown in Figure 4c or 4d, respectively. Clearly, present images do not bear much information on the overlying HY layer, not even if just to comment about its presence and homogeneity. Nor can be more information obtained by further increasing magnification, because image contrast cannot provide vertical resolution in the nanometer range.

Chemical analysis by EDX yielded results very similar to those reported in the paper by Morra et al., 2018: EDX spectra, both in the machined and microrough portion, are largely dominated by the peaks of titanium and show just a minor contribution from oxygen and carbon. Basically, they do not provide enough information to confirm the chemistry of the surface layer or to speculate on its chemical nature. They bear, however, an important information: The thickness of the hydrophilic surface layer that promotes the interfacial events shown in Figures 2 and 3 should be indeed in the nanometer range, hence the failure of EDX, whose sampling depth is a few micrometers, that is, three order of magnitude thicker, to clearly detect it.

### 3.3 X‐ray photoelectron spectroscopy

Chemical composition data obtained on probing the surface of tested implants by XPS is reported in Table 1. The most obvious observation is that titanium is not included among elements making up the surface chemistry of the tested Ti Grade 4 implant. No evidence of a role of the underlying topography (i.e., machined vs. microrough) on surface stoichiometry can be inferred from obtained data. The resulting information is that an organic overlayer, whose thickness is bigger than the XPS sampling depth (about 5 nm), homogenously covers the underlying implant.

**Table 1:** Surface composition (% at.) of the tested implants, as detected by XPS analysis (mean and std of three measurements)

Sample	O	N	C
Lot 20173288 machined portion	33.8 ± 0.4	8.6 ± 0.2	57.6 ± 0.6
Lot 20173288 microrough portion	32.4 ± 0.5	8.3 ± 0.2	59.3 ± 0.5
Lot 20174409 machined portion	33.1 ± 0.5	8.6 ± 0.2	58.3 ± 0.6
Lot 20174409 microrough portion	32.7 ± 0.5	8.5 ± 0.3	58.8 ± 0.5

**Figure 5:**
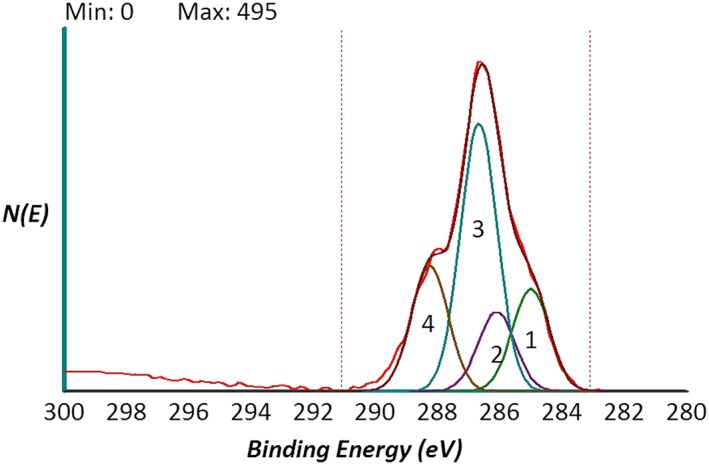
High‐resolution C1s peak of the tested implant surface obtained by XPS analysis. The peak is curve fitted by four components: 1 = C–C, C–H; 2 = C–N; 3 = C–O; 4 = C=O, N–C=O, and O–C=O following Shard et al. (1997)

High resolution C1s, O1s, N1s peaks were obtained to gather more information on the molecular structure of the surface layer. Again, no difference between data obtained in the different sections of the implants were recorded. The C1s peak was deconvoluted according to literature procedures to extract information on the carbon chemical environment. Results are shown in Figure 5. Following Shard et al. (1997), the experimental curve was fitted by four components located at 285.00 eV (C–C, C–H), 286.15 eV (C–N), 286.50 eV (C–O), and 288.10 eV (C=O, N–C=O, and O–C=O functionalities). Table 2 reports results of curve fitting and percent area occupied by the different components. The experimental C1s peak can be fitted very well using components expected from HY, confirming the molecular structure of the surface‐linked layer. The peak is largely dominated by the carbon‐single bond to oxygen component, that is, by polar C‐O functionalities and by the hydrophilic chemical moiety involving hydroxyl linked to carbon. Together with the contribution of the HY carboxyl group, these functionalities make HY a highly hydrophilic molecule and present XPS data confirm that they are maintained in the surface‐coupling process. Shortly, detailed analysis of the carbon chemical environment on the implant surface renders the image of a highly hydrophilic, water‐interacting milieu, providing the molecular basis for the macroscopic physical events captured in Figures 2 and 3.

**Table 2:** Results of curve fitting according to Shard et al. (1997) of the C1s peak shown in Figure 5. FWHM (Full Width at Half Maximum)

Component no.	Comment	Area	Area, %	Position	Intensity	FWHM
1	C–C, C–H	228	17.8%	285.01	153	1.40
2	C–N	177	13.8%	286.11	119	1.40
3	C–O	598	46.6%	286.68	401	1.40
4	C=O, N–C=O, O–C=O	280	21.8%	288.16	188	1.40

### 3.4 Atomic force microscopy

**Figure 6:**
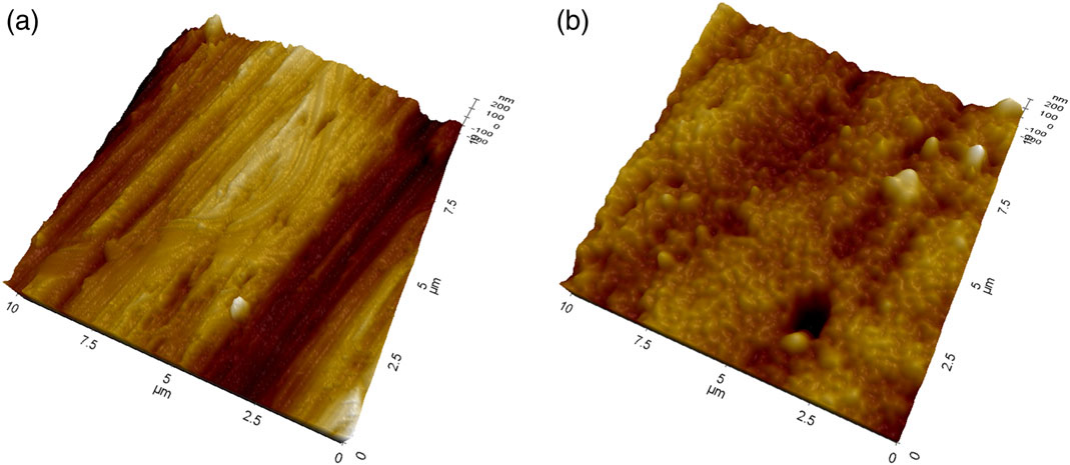
Atomic force microscopy three‐dimensional views of 10 × 10‐μm areas of tested Ti disks. (a) machined surface, not coated; (b) machined surface, HY coated

**Figure 7:**
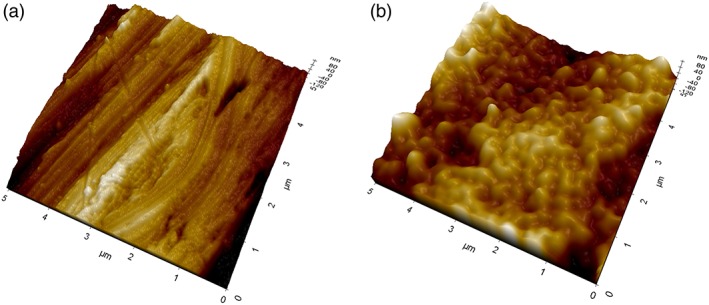
Atomic force microscopy three‐dimensional views of 5 × 5‐μm areas of tested Ti disks. (a) machined surface, not coated; (b) machined surface, HY coated

**Figure 8:**
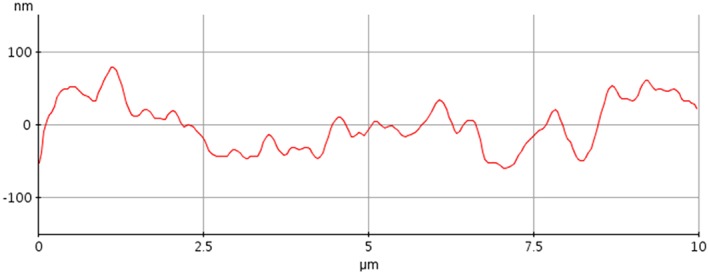
Atomic force microscopy height profile obtained by drawing a 10‐μm line in Figure 6b

Supplementary information on the surface structure were obtained by analysis of machined disks uncoated and subjected to the same surface modification treatment as implants. Main results from noncontact AFM analysis are reported in Figures 6 and 7. Figure 6a shows a 10 × 10‐μm field of view obtained on the machined surface (not coated). The imaged area has a similar size as compared with that shown in Figure 4a (15 × 10 μm). In Figure 6a, the main features are the already discussed parallel grooves due to machining. Moving to Figure 6b, that is, to the coated surface, thus the same subject shown in Figure 4a, a significantly different picture is observed. While the presence of the parallel grooves can still be inferred, a submicrometer bumped structure can be clearly detected. This is even more evident in Figure 7, comparing 5 × 5‐μm fields of view of the machined (a) and coated (b) surface (that is, to be compared with the SEM view of Figure 4b). In agreement with XPS findings, the nanotextured structure due to the coating covers homogenously the surface, that is, the underlying Ti does not “show through” (else, some Ti would be detected in the XPS spectra). Note that the vertical scale of the image is of the order of some tens of nanometers. Figure 8 is a height profile obtained by drawing a 10‐μm line in Figure 6b. Bumps range from a few nanometers to a few tens of nanometers. It is actually the exceptional vertical resolution of the AFM technique that allows to imagine topography details of the HY overlayer through nanoroughness contrast.

### 3.5 *ζ*‐potential measurement

Eletcrokinetic effects at the solid–liquid interface were evaluated through *ζ*‐potential measurement. In particular, as described in Section 2, the streaming current was measured as a function of pH, in the 2.8 to 8.2 range, in 1‐mM KCl solution.

**Figure 9:**
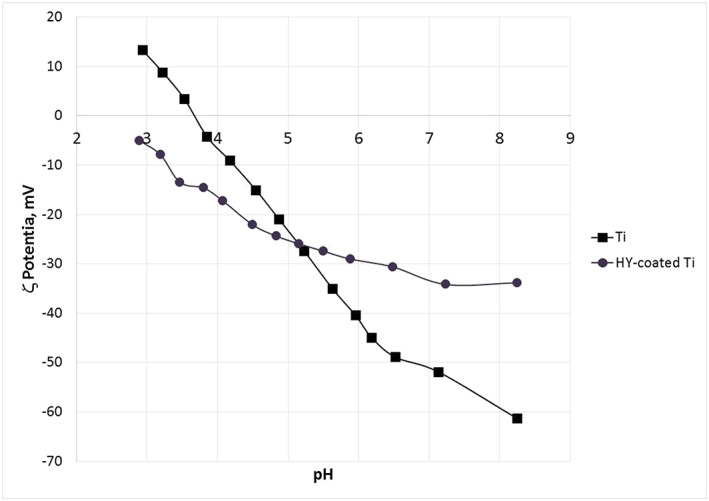
ς potential versus pH graphs, obtained by streaming current measurement in 1‐mM KCl solution, of Ti and HY‐coated Ti samples

Results are shown in Figure 9. In the case of Ti, the pH scan yields a curve that moves from positive *ζ*‐potential values at low pH to negative pH values at high pH, crossing the isoelectric point at 3.67. Hence, Ti is negatively charged at physiological pH. This behavior is qualitatively consistent with results obtained by Roessler, Zimmermann, Scharnweber, Werner, and Worch (2002) on differently oxidized TiAlV and Ti samples. Of relevance for the present discussion, the detected *ζ* potential versus pH relationship is fairly typical of a very weak acid–base interfacial activity (Luxbacher, 2014), in the sense that it is not dictated by acid–base dissociation of chemical functionalities, but mostly by adsorption of ions contained in the solution (OH^−^, H_3_O^+^, Cl^−^, K^+^) as a function of pH. At low pH, the concentration of H_3_O^+^ is high, and its adsorption at the solid/liquid interface makes the surface charge positive. At high pH, OH^−^ dominates and (together with Cl^−^ adsorption, as suggested by Roessler et al., 2002) surface charge turns negative. HY‐coated Ti shows a different behavior: First of all, no isoelectric point is detected, that is, the interfacial potential induced by the surface charge is negative within the whole scanned pH range. Then, at high pH, the measured negative potential tends to plateau, contrary to the monotonous decrease detected on Ti. Taken together, these data indicate that the interfacial charge behavior is no longer dictated by pH‐dependent adsorption of ions from the solution, but by actual acid–base chemical functionalities operating at the solid/liquid interface (Luxbacher, 2014) as a function of pH. In particular, the surface shows an acidic behavior, that is, the *ζ* potential versus pH curve is dominated by the pH‐dependent dissociation of acidic groups yielding a negatively charged surface. Hence, the detected behavior is consistent with the polyanionic nature of HY and with the pH‐dependent dissociation of the carboxyl group contained in the D‐glucuronate moiety of its repeating unit.

## 4 DISCUSSION

HY nanoengineered dental implant surfaces recently reached clinical practice (Morra et al., 2018). HY is a linear polysaccharide consisting of the repeating disaccharide unit N‐acetyl‐D‐glucosamine‐D‐glucuronate, linked by β1‐4 and β1‐3 linkages. It is involved with a huge number of cellular processes related to tissue regeneration and repair (Abatangelo & Weigel, 2000; Evered & Whelan, 1989; Kennedy, Phillips, Williams, & Hascall, 2002; Laurent, 1998). There is a significant background on its use in the surface modification of medical materials and devices (Morra, 2005). In the spirit of biomolecular modification of implant surfaces (Puleo & Nanci, 1999), the presentation of HY molecular motifs at the implant/tissue interface could promote healing mechanisms not provided by the Ti interfacial chemistry.

Beside specific interactions, HY is also a paradigmatic hydrophilic molecule (Toole, 2000). The present work shows the impact of surface engineering by HY on wetting properties of Ti dental implants. The wetting behavior of the basic device of dental implantology, the titanium fixture, is bounded by native and induced aspects. The most important native feature is the chemical nature of Ti that leads to a high‐energy titanium oxide surface, as described in Section 1. Induced aspects involve the hierarchical structure of threads, screw pitch, concave grooves generated by sandblasting (in the range of 5 to about 70 μm), and pores of different shapes created by acid etching (in the range of several hundred nanometers up to 3 μm). This combination naturally leads to hydrocarbon adsorption (Att et al., 2009; Choi et al., 2017a, 2017b) and a wetting behavior that, as explained in basic teaching of wettability science (Cassie & Baxter, 1944, Dettre & Johnson Jr, 1964, Wenzel, 1936a, 1936b), is magnified by topographic details, leading to hydrophobic implant surfaces and large contact angle hysteresis on aging, as nicely described by Rupp et al. (2014). Within these boundaries, prevention of hydrocarbon adsorption by wet storage (Baier & Meyer, 1988) or removal of adsorbed hydrocarbons by discharge techniques just before usage (Canullo et al., 2016; Choi et al., 2017a, 2017b) stop or reverse the natural trend and provide high‐energy surfaces whose hydrophilicity is further enhanced by capillary wicking into grooves and pores.

Surface nanoengineering bypasses the boundaries of the surface chemistry of titanium, while keeping the surface topography motifs developed in years of basic studies and clinical practice (Figure 4; Rodriguez y Baena, Rizzo, Manzo, & Lupi, 2017; Wennerberg & Albrektsson, 2010). The chemical data reported into this work (EDX, XPS, and *ζ* potential) display a different picture of the surface chemistry of the nanoengineered basic device of dental implantology. The molecular structure of the HY layer does not belong to high‐energy surfaces; hence, it does not promote hydrocarbon adsorption, and it keeps its chemical identity on aging (Figure 5). It presents at the solid/liquid interface a high density of hydrophilic moieties, that is, polar carbon–oxygen bonds, hydroxyl groups, and carboxylate functionalities (Figure 5 and Table 2). The latter control charge behavior at the solid/liquid interface as shown in Figure 9, contrary to Ti where the behavior is controlled by ions in the solution. The markedly hydrophilic nature of the HY‐coated Ti surface combines with the hierarchical induced microstructure (Figure 4) promoting wicking and capillary rise (Figures 2 and 3) notwithstanding dry aging.

The present work shows detailed surface chemistry description of a novel dental implant for the first time in clinical use. The implant surface combines the recognized benefits of hybrid microrough topography with those expected from hydrophilic surfaces, with the potential role of the molecular structure of HY in tissue regeneration mechanisms. From a surface technology point of view, it is a significant step forward. Actual benefits for the clinical practice must confront with the very high success rate of “traditional” implant surfaces. The quoted clinical trial (Morra et al., 2018) showed no significant differences between HY‐coated and uncoated microrough surfaces with respect to marginal bone loss, in routine clinical practice, both provided optimal healing in the different clinical situations. In the same vein, a 6.5‐year radiological follow‐up evaluation of marginal bone loss around early loaded SLA and SLActive implants provided similar successful clinical results (Şener‐Yamaner, Yamaner, Sertgöz, Çanakçi, & Özcan, 2017). Although “the surface wettability of biomaterials determines the biological cascade of events at the biomaterial/host interface” …. (Rupp et al., 2014), clinical evidence suggests that as far as the host healing machinery works properly, differences in wetting behavior, on the long term, not necessarily improve further an already optimal clinical outcome. Results of the present work show that almost boundless progress of surface properties is achievable by surface engineering of dental implants of clinical use compared with the comparatively limited physical‐chemistry boundaries of titanium surfaces, aimed at opening new and faster healing pathways in compromised cases. In vitro and preclinical data largely support a role for surface hydrophilicity in periimplant bone regeneration and healing (Buser et al., 2004; Gittens et al., 2013; Morra et al., 2009; Park et al., 2012; Schwarz et al., 2007; Wennerberg et al., 2014) and provide the ethical basis to put all the instruments of surface science at the service of dental implantology.

## 5 CONCLUSIONS

In conclusion, data presented in this work show that nanoengineering of dental implant surfaces by HY results in permanently hydrophilic surfaces, overcoming time‐dependent progression towards hydrophobicity typical of conventional titanium surfaces due to adsorption of ubiquitous hydrocarbons from the atmosphere. Even after 1‐year dry storage on the shelf, the hydrophilic HY molecular layer permanently linked to the implant surface promotes strong capillary rise within the implant threads. Advanced surface analysis techniques endowed with nanometer‐investigation capability such as XPS, AFM, and *ζ* potential show a clear picture of the implant surface chemistry as opposed to that of conventional Ti surfaces.

## FUNDING INFORMATION

The study was supported by Nobil Bio Ricerche srl, the profit company that executes the surface treatment process.

## CONFLICT OF INTEREST

Marco Morra and Clara Cassinelli own shares of the funding company Nobil Bio Ricerche srl. Elisa Torre and Giorgio Iviglia are employees of Nobil Bio Ricerche srl.
